# Unique proximal tubular cell injury and the development of acute kidney injury in adult patients with minimal change nephrotic syndrome

**DOI:** 10.1186/s12882-017-0756-6

**Published:** 2017-11-28

**Authors:** Yoshihide Fujigaki, Yoshifuru Tamura, Michito Nagura, Shigeyuki Arai, Tatsuru Ota, Shigeru Shibata, Fukuo Kondo, Yutaka Yamaguchi, Shunya Uchida

**Affiliations:** 1 Department of Internal Medicine and Central Laboratory, Teikyo University School of Medicine, 2-11-1 Kaga, Itabashi-ku, Tokyo, Japan; 2 Department of Internal Medicine, Teikyo University School of Medicine, 2-11-1 Kaga, Itabashi-ku, Tokyo, Japan; 3 Department of Pathology, Teikyo University School of Medicine, 2-11-1 Kaga, Itabashi-ku, Tokyo, Japan

**Keywords:** Acute kidney injury, Minimal change nephrotic syndrome, Proximal tubule, N-acetyl-β-D-glucosaminidase, Vimentin

## Abstract

**Background:**

Adult patients with minimal change nephrotic syndrome (MCNS) are often associated with acute kidney injury (AKI). To assess the mechanisms of AKI, we examined whether tubular cell injuries unique to MCNS patients exist.

**Methods:**

We performed a retrospective analysis of clinical data and tubular cell changes using the immunohistochemical expression of vimentin as a marker of tubular injury and dedifferentiation at kidney biopsy in 37 adult MCNS patients. AKI was defined by the criteria of the Kidney Disease: Improving Global Outcomes (KDIGO) Clinical Practice Guidelines for AKI.

**Results:**

Thirteen patients (35.1%) were designated with AKI at kidney biopsy. No significant differences in age, history of hypertension, chronic kidney disease, diuretics use, proteinuria, and serum albumin were noted between the AKI and non-AKI groups. Urinary N-acetyl-β-D-glucosaminidase (uNAG) and urinary alpha1-microglobulin (uA1MG) as markers of tubular injury were increased in both groups, but the levels were significantly increased in the AKI group compared with the non-AKI group. The incidence of vimentin-positive tubules was comparable between AKI (84.6%) and non-AKI (58.3%) groups, but vimentin-positive tubular area per interstitial area was significantly increased in the AKI group (19.8%) compared with the non-AKI group (6.8%) (*p* = 0.011). Vimentin-positive injured tubules with tubular simplification (loss of brush-border of the proximal tubule/dilated tubule with flattening of tubular epithelium) were observed in the vicinity of glomeruli in both groups, suggesting that the proximal convoluted tubules were specifically injured. Two patients exhibited relatively severe tubular injuries with vimentin positivity and required dialysis within 2 weeks after kidney biopsy. The percentage of the vimentin-positive tubular area was positively correlated with uNAG but not with uA1MG in the non-AKI group.

**Conclusions:**

Proximal tubular injuries with increased uNAG exist in MCNS patients without renal dysfunction and were more severe in the AKI group than they were in the non-AKI group. The unique tubular injuries probably due to massive proteinuria might be a predisposing factor for the development of severe AKI in adult MCNS patients.

## Background

Acute kidney injury (AKI) is a well-known complication of minimal change nephrotic syndrome (MCNS) that occurs in 25 to 40% of adult patients [1–4] and is typically reversible with a favourable response to corticosteroid treatment [1]. However, a small number of MCNS patients with severe AKI with or without the requirement of dialysis has been reported, and these patients develop irreversible renal dysfunction [1, 5].

It was suggested that AKI in patients with MCNS is the result of severe plasma volume depletion due to low plasma oncotic pressure. However, the absolute and relative blood volumes and renal plasma flow were well preserved in most patients with nephrotic syndrome [6]. Recent evidence has demonstrated that mechanisms intrinsic to the kidney contribute to sodium retention and edema formation in nephrotic syndrome, including activation of epithelial sodium channel (ENaC) by aberrantly filtered proteases [7]. The pathogenesis of severe AKI remains uncertain and is suggested to include (1) ischaemic renal injury [2, 8], (2) tubular obstruction by surrounding interstitial edema [9], (3) redistribution of renal blood flow from cortical to juxtaglomerular nephrons [10] and (4) decrease in capillary filtration coefficient (Kf) [3, 6, 11]. In addition to these functional and structural alterations in the kidney, proteinuria has been proposed to induce tubular cell injury and apoptosis [12–14]. AKI generally lasts longer in MCNS patients than it does in patients with ischaemic acute tubular necrosis (ATN) (average duration of 7 weeks vs. 10 to 14 days) [8], and reduction of proteinuria is indispensable for recovery from AKI. However, it is unlikely that massive proteinuria itself is the exclusive mechanism of severe AKI because massive proteinuria is a constant phenomenon in all MCNS patients. Thus, multiple factors may be involved in the pathophysiology of developing severe AKI in MCNS.

Clear vacuoles or hyaline droplets, which represent resorbed protein, are commonly observed in tubular cells in MCNS patients [15]. However, it is not clear whether vacuoles or hyaline droplets indicate tubular injuries as pathological changes. Tubular lesions associated with acute tubular injuries were also reported in MCNS patients with severe AKI. However, given the lack of unified standard morphologic criteria for acute tubular injuries, it seems difficult to evaluate tubular injuries quantitatively in MCNS patients by light microscopic morphology.

In the present study, we attempted to investigate the mechanisms of AKI in MCNS patients and focused specifically on the expression of vimentin in the kidney tubules as a marker of tubular injury and dedifferentiation [16] and the expression of Ki67 as a marker of tubular cell proliferation or regeneration [17]. Then, the findings of vimentin expression in tubules and clinical parameters were compared between AKI and non-AKI groups at kidney biopsy.

## Methods

### Clinical characteristics of patients and definition of clinical parameters

The clinical records of adult patients (older than 18 years) who had the initial presentation of biopsy-proven MCNS at Teikyo University Hospital from January 2008 to December 2015 were retrospectively reviewed. All patients received corticosteroid treatment several days before or after kidney biopsy. History of hypertension was defined as patients who used an anti-hypertensive drug, and hypertension was defined as systolic blood pressure readings of ≥140 mmHg or diastolic blood pressure readings of ≥90 mmHg. Hypotension was defined as systolic blood pressure readings of ≤90 mmHg or diastolic blood pressure readings of ≤60 mmHg.

Laboratory parameters at kidney biopsy included serum creatinine (Cr), serum albumin, urinary protein excretion, haematuria (≥ 5 red blood cells per high power field), urinary N-acetyl-β-D-glucosaminidase (uNAG), urinary alpha1-microglobulin (uA1MG), and urinary Cr excretion. The estimated glomerular filtration rate (eGFR) was calculated using the revised serum Cr–based Japanese equation [18]. The presence of chronic kidney disease (CKD) was defined by an eGFR <60 mL/min/1.73 m^2^ [19]. All of the urinary proteins were expressed as the ratio-to-Cr to correct variations in urine concentration among individuals. No accepted criteria are available regarding an abnormal amount of uNAG and uA1MG expressed as the ratio to Cr. Thus, according to the reported values, the abnormalities were defined as uNAG >9.1 IU/gCr and uA1MG > 25.8 mg/gCr, respectively [20]. Complete remission (CR) of nephrotic syndrome was defined as a daily urinary protein excretion of <0.3 g/gCr. The time to CR after corticosteroid-based therapy and the serum Cr at CR were recorded.

Post-renal AKI was excluded by abdominal echography in all patients. AKI was defined, and severity was staged based on the criteria for serum Cr of the Kidney Disease: Improving Global Outcomes (KDIGO) Clinical Practice Guidelines for AKI [21]. At kidney biopsy, AKI was defined as any of the following: an absolute increase in serum Cr ≥ 0.3 mg/dl within 48 or increase in serum Cr to ≥1.5 times the baseline value, which is known or presumed to have occurred within the prior 7 days. Baseline serum Cr was defined as the lowest serum Cr value available prior to the date of kidney biopsy or the value of serum Cr when it rapidly decreased.

### Histological characteristics

Kidney biopsy tissue specimens including at least 10 glomeruli were examined by light microscopy, immunofluorescence and electron microscopy, and all the patients were diagnosed with MCNS [15]. ATN and tubular lesions associated with acute tubular injuries [5, 22] were assessed by the presence of tubular necrosis/detachment of tubular cells from the basal lamina and tubular simplification (absence or thinning of brush-border of the proximal tubule/dilated tubule with flattening of tubular epithelium), respectively. The presence of vacuole/hyaline droplets in tubular cells, proteinaceous cast, severe interstitial edema with tubular collapse, interstitial inflammation/fibrosis and arteriosclerosis or arteriolosclerosis was also examined.

For immunohistochemical staining, 3-μm-thick, 10% formalin fixed, paraffin sections were deparaffinized, microwaved with citrate buffer for antigen retrieval, and incubated with primary antibodies for 1 h at 37 °C. Sections were treated with 3% H_2_O_2_ for 10 min to inactivate endogenous peroxidase activity. Sections were incubated with the primary antibodies, including murine monoclonal anti-vimentin antibody (clone V9, Sigma Aldrich, St Louis, MO, USA) or murine monoclonal anti-human Ki67 antibody (clone MIB-1, Dako Denmark A/S, Glostrup, Denmark); reacted with Histofine Simple Stain MAX PO® (Nichirei Bioscience Inc. Tokyo, Japan); and visualized using a peroxidase-diaminobenzidine system. Nuclei were counterstained with haematoxylin. For control sections, primary antibodies were omitted or replaced with normal mouse serum. Signals in control sections were negative or negligible.

For the semi-quantification of immunohistochemical staining of vimentin, the entire slide images of each tissue were scanned at ×20 magnification using a Nano Zoomer 2.0 HT® (Hamamatsu Photonics K.K., Hamamatsu, Japan). The ratio of the area of vimentin-positive tubules relative to the area of the tubulointerstitium of the entire cortex and outer stripe of outer medulla was calculated by using the Aperio ImageScope software (Leica Biosystems, Nussloch, Germany). A vimentin-positive tubule was defined as one in which any tubular cells expressed vimentin within the tubule. The measurements were performed after exclusion of glomeruli and major vessels, and the results were presented as the percentage of vimentin-positive tubular areas.

### Statistical analysis

Values of continuous variables were reported as the means ± standard deviation (SD). Student’s *t* test and Mann-Whitney *U* test were performed to compare values between groups as appropriate. Categorical variables were described as a percentage and were compared using the Fisher’s exact test. Correlations among clinical biomarker values, and vimentin staining results were evaluated by Pearson correlation coefficient and Spearman’s rank correlation coefficients where appropriate. Statistical significance was assumed at *P* < 0.05. Statistical analyses were performed using GraphPad Prism7 (GraphPad Software Inc., San Diego, CA, USA).

## Results

### Characteristics of the clinical parameters in AKI and non-AKI groups

Thirty-seven patients with MCNS (22 men, 15 women) aged between 18 and 77 years (average age 44.3 ± 19.4 years) were evaluated. Table 1 presents the clinical and laboratory characteristics of all patients and patients with or without AKI at kidney biopsy. Thirteen patients (35.1%) met the criteria of AKI according to KDIGO Clinical Practice Guideline for AKI. No significant differences in age, gender, the presence of CKD and diuretics use were noted between AKI and non-AKI groups. Nonsteroidal anti-inflammatory drugs were not used in both groups. The incidence of a history of hypertension was not different between groups, but the AKI group exhibited increased systolic blood pressure compared with the non-AKI group. Two patients in the non-AKI group exhibited diastolic hypotension at kidney biopsy. All patients exhibited pitting edema.

**Table 1:** Comparison of the clinical characteristics in patients with and without AKI

Characteristics	All	AKI	Non-AKI
No of patients	37	13	24
Age (years)	44.3 ± 19.4	50.8 ± 21.0	40.8 ± 17.9
Gender (% male)	59.4	69.2	54.1
History of HT (%)	24.3	30.7	20.8
Hypotension (%)^a^	5.4	0	8.3
Systolic BP (mmHg)	133.2 ± 13.3	140.4 ± 12.2	129.4 ± 12.5*
Diastolic BP (mmHg)	79.6 ± 10.8	83.8 ± 9.1	77.5 ± 11.1
Presence of CKD (%)	8.1	15.3	4.1
Diuretics use (%)	64.8	76.9	58.3
Proteinuria (g/gCr)	9.01 ± 3.53	9.09 ± 3.97	8.97 ± 3.36
Hematuria (%)	16.2	15.3	4.1
uNAG (IU/gCr)	25.7 ± 15.4	33.0 ± 19.7	21.7 ± 11.1*
uA1MG (mg/gCr)	53.9 ± 31.1	72.9 ± 38.1	43.6 ± 31.5*
serum Cr (mg/dl)	1.15 ± 0.57	1.72 ± 0.62	0.84 ± 0.18***
eGFR (ml/min/1.73 m^2^)	62.4 ± 27.2	35.3 ± 18.1	76.2 ± 19.8***
Serum albumin (g/dl)	1.5 ± 0.4	1.4 ± 0.2	1.6 ± 0.4
Time to CR (days)	26.4 ± 26.7	43.0 ± 36.0	17.4 ± 14.0**
Serum Cr at CR (mg/dl)	0.93 ± 0.53	1.20 ± 0.83	0.78 ± 0.16*

Data expressed as the mean ± SD or proportions

*HT* hypertension, *BP* blood pressure, *CKD* chronic kidney disease, *uNAG* urinary N-acetyl-β-D-glucosaminidase, *uA1MG* urinary alpha1-microglobulin, *Cr* creatinine, *eGFR* estimated glomerular filtration rate, *CR* complete remission. ^a^Hypotension at kidney biopsy

* *P* < 0.05, ** *P* < 0.01 and *** *P* < 0.001 vs. AKI group were indicated

No significant differences in serum albumin, urinary protein excretion and presence of haematuria were noted between AKI and non-AKI groups. Serum Cr was increased, and eGFR was reduced in the AKI group compared with the non-AKI group. uNAG and uA1MG were increased in both groups, but these values were significantly increased in the AKI group compared with the non-AKI group.

Correlations between uNAG and uA1MG and biomarker values were examined (Table 2). uNAG was correlated positively with serum Cr and inversely with eGFR and serum albumin in all patients (*r* = 0.587, *p* < 0.001; *r* = −0.462, *p* = 0.003; r = −0.462, p = 0.003, respectively). uNAG was correlated positively with serum Cr in each group (AKI *r* = 0.714, *p* = 0.006; non-AKI *r* = 0.601, *p* = 0.001). uA1MG was correlated positively with serum Cr and urinary protein excretion and inversely correlated with eGFR and serum albumin in all patients (*r* = 0.456, *p* = 0.004; *r* = 0.388, *p* = 0.017; *r* = −0.489, *p* = 0.002; *r* = −0.437, p = 0.006, respectively). uA1MG was correlated positively with serum Cr in the non-AKI group (*r* = 0.503, *p* = 0.012) and inversely correlated with serum albumin in the AKI group (*r* = −0.843, *p* < 0.001). uNAG and uA1MG were not correlated with urinary protein excretion in each group.

**Table 2:** Correlations between uNAG and uA1MG and biomarker values in patients with and without AKI

	Total		AKI		Non-AKI	
	r	*P*	r	*P*	r	*P*
	uNAG					
Serum Cr (mg/dl)	0.587	<0.001	0.714	0.006	0.601	0.001
eGFR (ml/min/1.73 m^2^)	−0.462	0.003	−0.419	ns	−0.269	ns
Serum albumin (g/dl)	−0.462	0.003	−0.419	ns	−0.269	ns
Proteinuria (g/gCr)	0.288	ns	0.352	ns	0.261	ns
	uA1MG					
Serum Cr (mg/dl)	0.456	0.004	0.212	ns	0.503	0.012
eGFR (ml/min/1.73 m^2^)	−0.489	0.002	−0.221	ns	−0.392	ns
Serum albumin (g/dl)	−0.437	0.006	−0.843	<0.001	−0.252	ns
Proteinuria (g/gCr)	0.388	0.017	0.469	ns	0.374	ns

### Histological findings in AKI and non-AKI groups

**Fig. 1:**
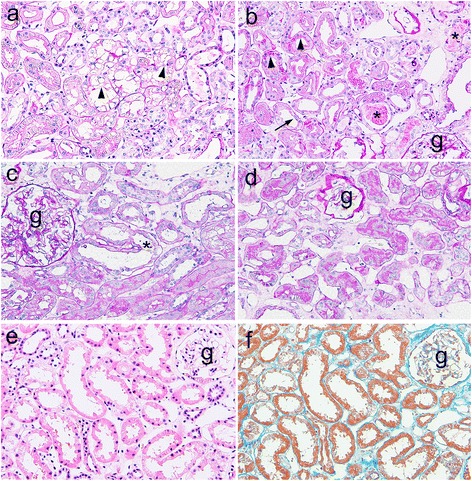
Representative findings of tubulointerstitial changes in MCNS patients. **a-f** Vacuoles (**a**, arrow heads), hyaline droplets (**b**, arrow heads) and proteinaceous casts (**b**, asterisks) are observed in tubules. Tubular simplification, including loss of brush-border of the proximal tubule (**b**, arrow) and dilated tubule with flattening of tubular epithelium, is also observed. **c** Tubular necrosis is focally observed in a patient in the AKI group (asterisk). **d** Severe interstitial edema due to the expansion of intertubular spaces and dilated peritubular capillaries with tubular collapses is noted. H & E staining (**e**) and Masson’s trichrome staining (**f**) in consecutive sections. Tubules with tubular simplification (**e**) are situated on the thin tubular basement membrane that appears to be normal and is not surrounded by an accumulation of collagen fibres, as assessed by Masson’s trichrome-positivity (**g**: glomeruli; **a-d**, PAS staining; original magnification ×200).

Kidney biopsy findings were summarized in Table 3. As reported previously [15], vacuoles (Fig. 1a) or hyaline droplets (Fig. 1b) in tubular cells were focally observed in most patients. Proteinaceous casts were frequently observed in both groups (Fig. 1b). Only rare tubules with evidence of necrosis and/or tubular cell detachment from the basal lamina were observed in 4 patients of the AKI group (Fig. 1c). Tubular simplification (loss of brush-border of proximal tubules/dilated tubule with flattening of tubular epithelium) was focally located in both groups (Fig. 1b), and the presence of tubular simplification was significantly increased in the AKI group compared with the non-AKI group (Table 3). Interstitial edema was often observed in both groups (Fig. 1b), and no significant difference in the existence of severe interstitial edema with tubular collapse (Fig. 1d) was noted between both groups. The presence of interstitial fibrosis or inflammation and arteriosclerosis or arteriolosclerosis did not differ between both groups (not shown). Atrophic tubules were sporadically observed in association with interstitial fibrosis in some patients; however, most tubules with tubular simplification in both groups were not surrounded by an accumulation of collagen fibres, as judged by Masson’s trichrome positivity in this study (Fig. 1e and f).

**Table 3:** Comparison of renal pathological data in patients with and without AKI

Kidney biopsy findings	All	AKI	Non-AKI
No of patients (%)	37	13	24
Vacuole/hyaline droplet	34 (98.1)	12 (92.3)	22 (91.6)
Proteinaceous cast	17 (45.9)	8 (61.5)	9 (37.5)
Severe interstitial edema^a^	6 (16.2)	3 (23.0)	3 (12.3)
Tubular necrosis/detachment	4 (10.8)	4 (30.7)	0 (0)*
Tubular simplification^b^	19 (62.1)	11 (84.6)	9 (37.5)*
Interstitial lesions^c^	6 (16.2)	4 (30.7)	2 (8.3)
Vascular lesions^d^	13 (35.1)	6 (46.1)	7 (29.1)
Vimentin + tubules	25 (67.5)	11 (84.6)	14 (58.3)
Vimentin + tubular areas^e^	11.3 ± 13.7	19.8 ± 18.3	6.8 ± 7.5*

^a^severe interstitial edema with tubular collapse

^b^loss of brush-border of the proximal tubule/dilated tubule with flattening of tubular epithelium

^c^interstitial inflammation/fibrosis

^d^arteriosclerosis or arteriolosclerosis

^e^mean percentage of vimentin-positive tubular areas

*P < 0.05 vs. AKI group was indicated

### Vimentin expression in tubular cells and its comparison with clinical parameters

**Fig. 2:**
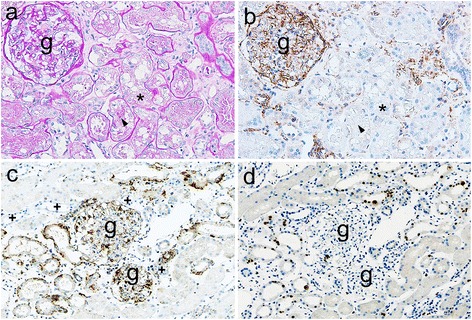
Representative findings of tubules in MCNS patients in the non-AKI group. **a-b**. PAS staining (**a**) and staining of vimentin (**b**) in consecutive sections. Mesangium and vessels are positive for vimentin, but tubules are negative for vimentin. Note, tubules with vacuoles (**a-b**, asterisks) and hyaline droplets (**a-b**, arrow head) do not express vimentin. **c-d** Staining of vimentin (**c**) and staining of Ki67 (**d**) in consecutive sections. Small groups of simplified tubules include cells with vimentin-positivity in basal or cytoplasmic patterns, which are in the vicinity of a glomerulus (**c**). + indicate the distal tubule from a morphological aspect (**c**). Ki67 is expressed in some tubular cells among the vimentin-positive tubules (**d**) (**g**: glomeruli; original magnification ×200).

**Fig. 3:**
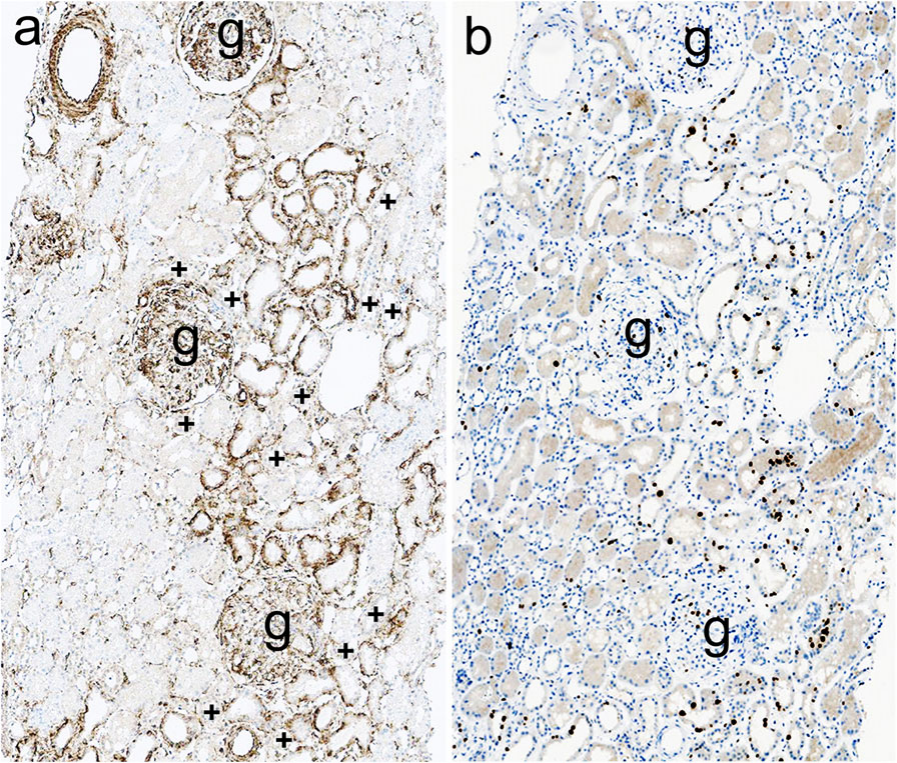
Representative findings of tubules in MCNS patients in the AKI group. **a-b** Staining of vimentin (**a**) or Ki67 (**b**) in consecutive sections. Mesangium and vessels are positive for vimentin, but approximately normally shaped tubules are negative for vimentin. Small groups of vimentin-positive tubules exhibiting mainly simplified morphology are observed in the vicinity of glomeruli (**a**). + indicate the distal tubule from a morphological aspect (**a**). Most Ki67-positive cells are observed in vimentin-positive tubules (**b**) (**g**: glomeruli; original magnification ×200).

Vimentin is observed in mesangium and blood vessels but not in tubule cells in normal human adult kidneys. In addition, vimentin expression in tubular cells indicated tubular injury or dedifferentiation [23]. In this study, mesangium and blood vessels were positive for vimentin (Fig. 2b and c). Most proximal tubular cells exhibiting vacuoles and hyaline droplets did not necessarily express vimentin (Fig. 2a,b), suggesting that these pathological changes are not associated with vimentin expression. In contrast, small groups of vimentin-positive tubules primarily exhibiting tubular simplification (loss of brush-border of the proximal tubule/dilated tubule with flattening of tubular epithelium) were often observed in the vicinity of glomeruli in both groups (Figs. 2c and 3a). Most of them were not surrounded by vimentin-positive fibroblastic cells (Figs. 2c and 3a). Vimentin-positive staining was observed in basal or cytoplasmic patterns in tubular cells but not in the distal tubular cells from a morphological aspect (Fig. 2c). The number of patients with vimentin-positive tubules was 11 (84.6%) out of 13 patients in the AKI group and 14 (58.3%) out of 24 patients in the non-AKI group, and no significant difference was noted between groups (*p* = 0.149) (Table 3). However, the percentage of vimentin-positive tubular areas was significantly increased in the AKI group compared with the non-AKI group (*p* = 0.011) (Table 3, Figs. 2c and 3a).

Ki67 was expressed in some tubular cells among the vimentin-positive tubules in both groups (Figs. 2c, d and 3a, b), indicating that some tubular cells were proliferating or regenerating to repair the damaged tubules not only in the AKI group but also in the non-AKI group.

**Fig. 4:**
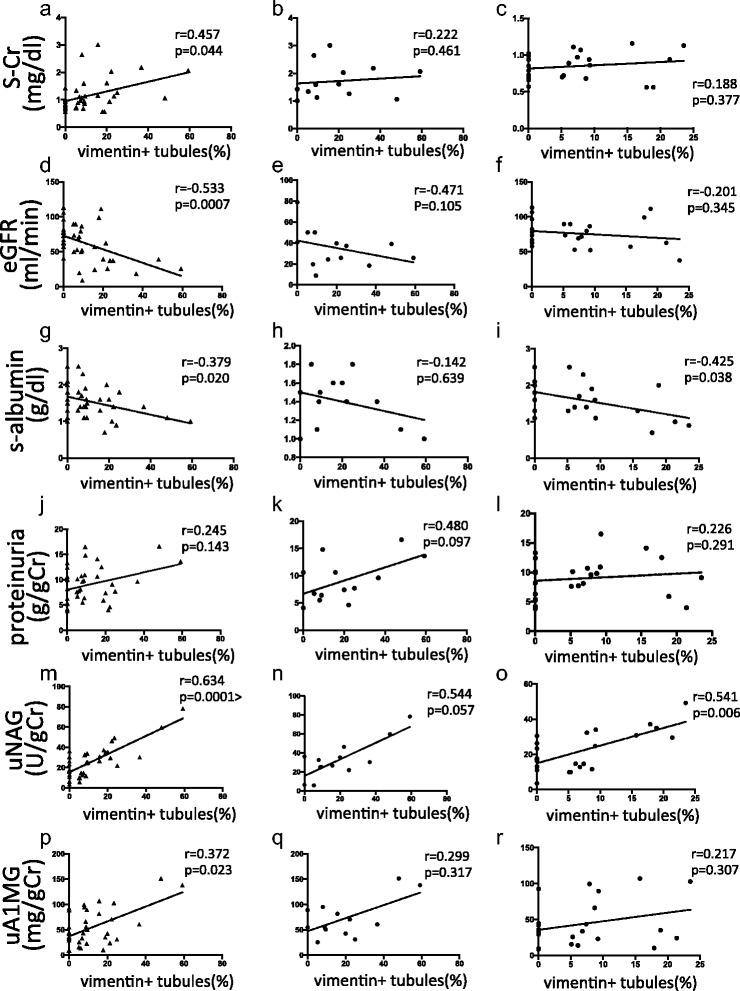
Correlations between the percentage of vimentin-positive tubular areas and biomarker values. Total patients (**a**, **d**, **g**, **j**, **m** and **p**) and patients with (**b**, **e**, **h**, **k**, **n** and **q**) or without AKI (**c**, **f**, **i**, **l**, **o** and **r**). The percentage of vimentin-positive tubular areas vs. serum Cr (s-Cr) (**a**, **b** and **c**), eGFR (**d**, **e** and **f**), serum albumin (s-albumin) (**g**, **h** and **i**), proteinuria (**j**, **k** and **l**), uNAG (**m**, **n** and **o**) and uA1MG (**p**, **q** and **r**)

The percentage of vimentin-positive tubular areas in all patients was significantly and positively correlated with serum Cr, uNAG and uA1MG and inversely correlated with eGFR and serum albumin (Fig. 4). The percentage of vimentin-positive tubular areas was correlated positively with uNAG and inversely correlated with serum albumin in the non-AKI group (Fig. 4).

### Clinical outcomes

All patients started corticosteroid therapy within a few days before or 7 days after kidney biopsy. After treatment, all patients achieved CR. The mean time to CR was significantly longer in the AKI group (43.0 ± 36.0 days) than it was in the non-AKI group (17.4 ± 14.0 days) (Table 1).

Two patients with vimentin-positive tubules in the non-AKI group developed AKI within 7 days after kidney biopsy. However, renal dysfunction was mild (AKI stage 1: 1.5–1.9 times baseline of serum Cr), and the baseline serum Cr levels were restored within 2 weeks. Two patients without apparent vimentin-positive tubules in the AKI group exhibited relatively mild AKI, with the peak serum Cr averaging 1.22 mg/dl. In contrast, 11 patients with vimentin-positive tubular damage exhibited an average peak serum Cr of 3.17 ± 2.06 mg/dl. Two patients in the AKI group underwent haemodialysis due to oliguria at 10 days and 12 days after kidney biopsy and underwent haemodialysis for 8 weeks and 11 weeks, respectively. The same 2 patients exhibited 2.18 mg/dl and 1.06 mg/dl serum Cr at kidney biopsy and 36.7 and 48.0% vimentin-positive tubular areas, respectively.

A recovery of serum Cr was observed in most patients in the AKI group, but the serum Cr levels at the time of CR in the AKI group remained significantly increased compared with the non-AKI group (Table 1). Serum Cr in 2 patients did not return to baseline levels at the last follow-up (more than 1 year). One patient experienced a peak level of ≥3.0 times baseline of serum Cr (AKI stage 3), and another patient required temporary dialysis therapy (AKI stage 3).

## Discussion

One-third of our adult MCNS patients exhibited AKI at kidney biopsy, which was consistent with previous reports [1–4]. MCNS patients with AKI are characterized by increased urinary protein excretion, reduced serum albumin [1, 2, 5, 8], older age and hypertension at presentation [1] compared with patients without AKI. In this study, patients in the AKI group exhibited increased systolic blood pressure compared with the non-AKI group. However, in contrast to previous reports, the other parameters were similar in both groups, except for the uNAG and uA1MG levels. uNAG and uA1MG were increased in both groups, but their levels were significantly increased in the AKI group compared with the non-AKI group, suggesting that proximal tubular injuries exist in MCNS patients even without AKI and may be more severe in the AKI group compared with the non-AKI group. A positive correlation was reported between urinary protein excretion and uNAG in idiopathic nephrotic syndrome [24, 25]. However, we did not observe the same correlation. The timing of data collection and renal function might have some influence on the present results.

ATN and tubular simplification were reported in MCNS patients with severe AKI [5]. In this study, focal tubular necrosis/focal detachment of tubular cells was only observed in the AKI group, but tubular simplification was noted not only in the AKI group but also in the non-AKI group. Most tubules with tubular simplification in this study were not atrophic tubules typically associated with interstitial fibrosis observed in adult patients [26]. Vimentin expression in tubules confirmed that tubular injuries exist in both groups, and the degree of vimentin-positive tubular injuries was significantly increased in the AKI group compared with the non-AKI group. Even in the non-AKI group, tubular injuries were sufficiently severe to be repaired by Ki67-positive proliferating cells. Unique vimentin-positive tubules with simplified morphology in both groups were mainly located in the vicinity of glomeruli, suggesting that these tubules represent the convoluted portion of proximal tubules, which must be exposed to abnormally filtered massive protein from a glomerulus. Proteinuria could induce apoptosis in proximal tubular cells [12, 13], and urinary protein from MCNS patients induces cellular injuries and apoptosis in a human proximal tubular cell line [14]. In addition, the straight portion of proximal tubules is more vulnerable to ischaemic changes compared with the convoluted portion of proximal tubules [27]. Thus, it is conceivable that proximal tubular injuries in the non-AKI group may be caused primarily by toxicity due to massive proteinuria but not by altered intrarenal haemodynamics.

In the present study, the percentage of the vimentin-positive tubular area was correlated with uNAG in all patients and the non-AKI group, separately. Thus, uNAG levels may reflect the degree of subclinical tubular injury in the non-AKI group. On the other hand, the percentage of the vimentin-positive tubular area was correlated with uA1MG in all patients but not in each group. The data suggest that uNAG may more directly reflect proximal tubular injuries rather than uA1MG in the non-AKI group. The percentage of the vimentin-positive tubular area was not correlated with urinary protein excretion but was correlated inversely with serum albumin in all patients and the non-AKI group probably because levels of urinary protein excretion at kidney biopsy do not necessarily reflect the degree of accumulated protein toxicity to tubular cells. Increased uNAG levels and reduced serum albumin may indicate the presence of unique tubular cell injuries in MCNS patients without renal dysfunction.

In this study, the tubular injuries at kidney biopsy were not intense even in the AKI group. In contrast to ATN in animal AKI models, the pathologic findings of ATN in human are generally limited to the focal tubulointerstitium and often subtle despite severe AKI. In this context, renal blood flow is thought to be sufficient to prevent ATN but not sufficient to maintain GFR [28]. The same may be true in MCNS patients with AKI. However, vimentin-positive tubular injuries might make patients susceptible to coexisting haemodynamic alterations. If severe haemodynamic alterations persist together with acute toxic tubular injuries, the kidney cannot sacrifice GFR to preserve tubular integrity and tubular regeneration, resulting in severe AKI with intense ATN. In this study, two patients in the AKI group exhibited relatively severe vimentin-positive tubular injuries followed by increased serum Cr. These patients required haemodialysis within 2 weeks after kidney biopsy. Thus, we postulated that severe persistent intrarenal haemodynamic changes might be superimposed on acute toxic tubular injuries in these patients. To prevent severe AKI and the progression to CKD in adult MCNS patients, prompt diagnosis and treatment are necessary in the present conditions.

Some limitations of the present study should be noted. First, the study design was a retrospective, single-centre investigation with a small number of patients. Second, there was a lack of repetitive kidney biopsy to evaluate the alteration of vimentin expression. Despite these limitations, the present study clearly demonstrated the significance of vimentin expression in proximal tubular cells in association with the development of AKI observed in MCNS.

## Conclusions

Unique proximal tubular cell injuries probably due to massive proteinuria often exist in MCNS patients with or without AKI. The uNAG levels may reflect the degree of subclinical tubular cell injury in the non-AKI group. Toxic tubular cell injuries with superimposition of altered intrarenal haemodynamics might contribute to the development of severe AKI in adult MCNS patients.

## Abbreviations

AKI: Acute kidney injury
ATN: Acute tubular necrosis
CKD: Chronic kidney disease
CR: Complete remission
Cr: Creatinine
eGFR: Estimated glomerular filtration rate
KDIGO: Kidney disease: Improving global outcomes
MCNS: Minimal change nephrotic syndrome
uA1MG: Urinary alpha1-microglobulin
uNAG: Urinary N-acetyl-β-D-glucosaminidase
